# Negative effect of varicocele on sperm mitochondrial dysfunction: A cross-sectional study

**DOI:** 10.18502/ijrm.v21i4.13271

**Published:** 2023-05-08

**Authors:** Mahshid Elahi, Vida Hojati, Mahmoud Hashemitabar, Mahsa Afrough, Hossain Mohammadpour Kargar, Maryam Dastoorpoor

**Affiliations:** ^1^Department of Biology, Damghan Branch, Islamic Azad University, Damghan, Iran.; ^2^Cellular and Molecular Research Center, Medical Basic Sciences Research Institute, Ahvaz Jundishapur University of Medical Sciences, Ahvaz, Iran.; ^3^Health Education Research Department, ACECR, Khuzestan, Ahvaz, Iran.; ^4^Department of Biostatistics and Epidemiology, Social Determinants of Health Research Center, Ahvaz Jondishapur University of Medical Sciences, Ahvaz, Iran.

**Keywords:** Mitochondria, ATP, Apoptosis, Varicocele, Male infertility.

## Abstract

**Background:**

Varicocele is an abnormal dilation and enlargement of the scrotal venous pampiniform plexus that impairs normal blood drainage and finally leads to infertility if not treated.

**Objective:**

This study aimed to figure out the impact of mitochondria status through the mitochondrial membrane potential (MMP) and adenosine triphosphate (ATP) assessment and its correlation with semen parameters to illuminate the impact of sperm mitochondria healthiness on normal sperm functionality.

**Materials and Methods:**

This analytical cross-sectional study was conducted with 100 men including 50 cases in the normozoospermic group (normal) and 50 in an infertile group with the non-varicocelectomy operation (varicocele) referring to Infertility Research and Treatment Center, ACECR Khuzestan, Iran. Routine semen analysis was performed according to World Health Organization guidelines, DNA fragmentation index, the MMP assay, ATP content, and apoptosis were carried out for all samples.

**Results:**

The results showed that the concentration, progressive motility, normal morphology, MMP, and ATP contents of sperm in varicocele were significantly lower than the normal group. In addition, the sperm DNA fragmentation index was significantly higher in the varicocele group in comparison with the normal group.

**Conclusion:**

Reduction in MMP and ATP contents, besides the loss of sperm parameters quality and increase in sperm DNA fragmentation, were seriously implicating sperm mitochondria dysfunctionality in varicocele men.

## 1. Introduction

Varicocele is the most common male factor of infertility with abnormal dilation and enlargement of the pampiniform plexus, which drains blood from each testicle to the single testicular vein. The decreased backflow of the vein is associated with hypoxia, which may be impaired testicular spermatogenesis, testicular volume, sperm parameters, and embryo implantation and pregnancy rate. Varicocele is the second most common cause of treatable infertility and occurs in around 15-20% of the general population. The incidence of varicocele increases with age (1). It accounts for 35% of primary infertility, and up to 80% of secondary infertile cases (2, 3). There is a controversy on the impact of varicocele before/after treatment of varicocele operation on sperm parameters and fertility. Numerous studies have shown that varicocelectomy not only improves semen parameters but also improves intra cytoplasmic sperm injection outcomes (4, 5).

The exact mechanism of varicocele remains unclear. The hypoplasia and insufficient growth of the testis in adolescence due to congenital/acquired valve defects, venous obstruction, and any anatomical variations contributed to its pathogenesis. This affected the ultrastructure of the testis, and testicular hormone dysfunction through high testicular temperature, scrotal hyperthermia, and blood reflux of the testis vein, and it may cause pain and swelling of the testis that needs a proper medical examination to cure infertility in varicoceles cases (6, 7). Recent studies have shown that there is a genetic downregulation in heat-shock proteins in varicoceles required for the neutralization of oxidative stress (OS). Testicular hyperthermia can induce the OS and increase reactive oxygen species (ROS) products (i.e., hydroxyl, peroxyl, hydroperoxyl radicals, superoxide, and nitric oxide) and induce sperm dysfunction (8, 9). An imbalance between ROS production and antioxidant protectant leads to OS and damage to lipids and proteins of the nucleic acids, induces sperm DNA and mitochondria nucleic acids fragmentation, damaged chromatin strands, and its crosslinks during the spermatogenesis and nuclear protamination in the varicocele's patients. (10, 11).

ROS production takes place mainly in mitochondria. Damage to sperm mitochondria, maybe, has correlation with varicocele and has a role in the pathogenesis of varicocele. Therefore, researchers recommend that it is better to examine the damage to semen parameters in infertile cases from a mitochondrial perspective. Sperm motility and, especially sperm functionality is highly dependent on ATP production through the glycolysis and mitochondrial oxidative phosphorylation system (OXPHOS) (12, 13), in addition to ROS production capacity. Therefore, before choosing the appropriate treatment method in ART, it is necessary to evaluate the rate of sperm DNA fragmentation, apoptosis, and also the health of sperm mitochondria as a criterion for the success of in vitro fertilization. In this study, the examination of the sperm mitochondria of normal and varicoceles individuals was aimed to investigate the relationship between the status of mitochondrial OXPHOS pathways in varicocele men by MMP and ATP contents assessment. And its relationship with semen parameters, apoptosis, and DNA fragmentation index (DFI) was evaluated.

## 2. Materials and Methods

### Study population 

In this analytical cross-sectional study, which was performed from July 2020 to November 2021, semen samples were collected from 100 men suffering from infertility, referring to infertility research and treatment, ACECR Khuzestan, Iran. Participants were divided into 2 groups, the first group consisted of 50 infertile men with varicocele and the second group included 50 normal without varicocele who were considered as a control group. Exclusion criteria: obese men (body mass index ≥ 30), people who underwent varicocele surgery, smokers, alcoholics, drug addicts, men with a history of liver disease, heart disease, tumor, testicular injury, testicular infection, kidney, and urinary tract infection, kidney stones, bladder stones, cancer, hepatitis, hyperlipidemia, hypertension, hernia, prostate damage, colitis, mumps, epilepsy, anemia, neurological problems, and people who had a high fever in the 3 months before sampling.

### Study protocol

Semen samples were collected after 2-5 days of sexual abstinence. Sperm parameters analysis was performed according to World Health Organization guidelines (14). After liquefaction (30-60 min), semen volume was measured by weighing the sample. Makler counting chambers for sperm count, routine manual procedures for estimating the proportion of progressive, nonprogressive, and immotile sperm, and Papanicolaou staining for morphology were used to perform routine semen analyses.

DFI assay was performed according to the manufacturer's instructions of the kit (Sperm DNA Fragmentation Assay, Dayan Zist co., Iran). At least 300 sperms were counted on each slide; haloed sperm was reported, while non-haloed sperm was determined for each sample as SDF percentage. The reference level was considered as ≥ 30% for the high DFI index and ≤ 15% for the low and moderate DFI index.

Phosphatidylserine (PS) is a marker for apoptosis that is exposed in the outer layer of plasma membrane in apoptotic cells. Annexin V is a protein that has the ability of binding PS in presence of Ca and labels the apoptotic sperms. According to the instructions of the kit (Annexin V-FITC Apoptosis Kit of Abnova Company, Cat#KA0714), semen samples were centrifuged at 1000 gr for 5 min, 2 times after liquefaction and prepared for swim-up according to World Health Organization protocol. The sediment motile sperm (1 × 10^6^/ml) were re-suspended by 500 μl of 1X binding buffer followed by 5 μl of Annexin V-FITC and 5 μl of propidium iodide (PI 50 μg/ml). The sperm samples were incubated at room temperature for 5 min in the dark. Then, the prepared samples were analyzed for Annexin V and Piusing FITC signal detector FL1 and the phycoerythrin emission signal detector FL2, respectively by flow cytometry (BD Face Callibur, Ex wavelength 488 nm).

According to the instructions of the kit (JC - 1 Mitochondrial Membrane Potential Assay kit of G Biosciences Company, Cat#786-1322), semen samples were centrifuged at 1000 gr for 5 min 2 times, after liquefaction and prepared for swim up. The sedimented motile sperm (1 × 10^6^/ml) were re-suspended with Ham's F10 medium. To prepare 2 μM JC - 1 working solution, 10 μl of 200 μM JC - 1 stock solution was added to 1 ml of mentioned culture medium and the sperms were incubated in an incubator (5% CO_2_, 37 C) for 15 - 30 min. To detect the inactivated MMP sperms, samples were immediately analyzed with a flow cytometer (BD Face Callibur) using a 488 nm excitation laser and green emission detector (FL1) and orange - red emission detector (FL2) for activated MMP sperms. The ratio of cells expressing red fluorescence was demonstrated in the R2 gate indicating the proportion of sperm with normal MMP activity.

Semen samples after liquefaction were prepared for swimming up to achieve the motile sperms, then 1 × 10^6^ motile sperms were frozen in liquid N2. The thawed samples were deproteinized following the kit instructions (De-proteinizing Sample Preparation Kit of Abnova Company, Cat#KA3716), and the measurement was performed according to the manufacturer's instructions (ATP assay kit of Abnova Company, Cat#KA0806) including ATP assay buffer, ATP probe, ATP convert, developer mix, and ATP standard. The concentration of ATP in the samples was estimated according to the standard curve and the results were expressed as nanomoles of ATP/106 spermatozoa using an existing filter 570 nm (BiotekElisa reader model ELX808).

### Ethical considerations

This cross-sectional study has been approved by the Ethics Committee for Humanities Research, Islamic Azad University Semnan Branch, Semnan, Iran. (Code: IR IR.IAU.SEMNAN.REC.1399.013). Written consent was obtained from the participants and a questionnaire containing personal information was completed for each person.

### Statistical analysis

Statistical analysis was performed using SPSS 22 (IBM Corp., Armonk, NY, USA). Descriptive statistics mean, and standard deviation was used in the study. The Kolmogorov-Smirnov test was used to examine the normality of the quantitative variables. The results of the test showed that the variables were abnormal. Therefore, a nonparametric test was adopted to analyze the data. The Mann-Whitney U test was used to compare the statistical differences between 2 groups. The Spearman correlation was used to analyze the correlations between DFI, MMP, ATP, apoptosis, and semen parameters. The significance level was set at p < 0.05.

## 3. Results

Among 500 men included in the current study, 100 semen samples including 50 varicocele and 50 normal samples by mean age between 27-50 yr were selected in the present study. The mean of sperm parameters, DFI, MMP, ATP contents, and apoptosis in normal and varicoceles groups were shown in table I. Data were presented as mean ± SE and the significance level was considered p < 0.001. Sperm concentration in varicocele was significantly lower than in normal men (p < 0.001). Also, the percentage of sperm motility in varicocele was significantly lower than in the normal group (p < 0.001). The percentage of normal sperm morphology in varicocele was significantly lower than in normal men (p < 0.001) as shown in table I.

**Figure 1:**
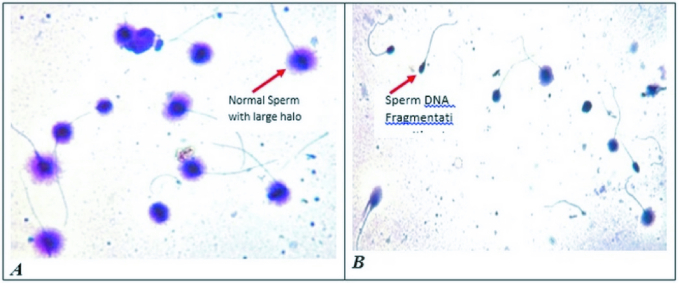
Photo by microscope during Sperm DNA Fragmentation analysis. Sperm DNA fragmentation in 2 groups A) normal and B) varicocele group.

The result of one sample in each group was shown in figure 1. The percentage of sperm DNA fragmentation in varicocele was significantly higher than the normal group (p < 0.001) (Table I). The relationship between sperm parameters and the percentage of sperm DNA damage in normal and varicocele was investigated, and the results were shown in table II. There was a significant negative relationship between sperm concentration (r = -0.508, p < 0.001), motility percentage (r = -0.370, p < 0.001), and sperm morphology (r = -0.610, p < 0.001) with DFI.

**Figure 2:**
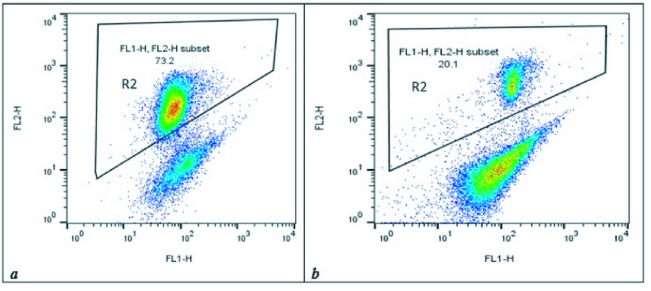
The flow cytometry analysis was performed by JC1 staining to demonstrate the red day conjugated ratio in sperm normal activated mitochondria in the R2 gate in the control group and varicocele group. The ratio of red fluorescent dye indicating the index of normal mitochondrial membrane potential (MMP) in 2 experimental groups. The mean ratio of cells expressing red fluorescence was obtained (a) MMP of the control group (R2 = 82.8 ± 15.1). (b) MMP of varicocele group (R2 = 38.2 ± 10.4).

MMP assay was performed by JC1 staining to detect sperm-active mitochondria between the 2 groups. The percentage of the accumulated red-florescent day in activated sperm mitochondria was estimated in the R2 gate, which represented the result of one sample of each group in figure 2. The mean of MMP in varicocele was approximately 2-fold lower than the normal group (p < 0.001) (Table I). The relationship between sperm parameters and MMP percentage in normal and varicocele was investigated, and the results were shown in table II. A significant positive relationship between sperm concentration (r = 0.805, p < 0.001), motility percentage (r = 0.679, p < 0.001), and normal sperm morphology (r = 0.712, p < 0.001) with MMP was observed. There was also a significant negative relationship between the percentage of DNA fragmentation and MMP (r = -0.417, p < 0.001). The level of ATP contents in varicocele (0.98 ± 0.08) was significantly lower than the normal group (1.3 ± 0.196), table I. The relationship between sperm parameters and ATP percentage in normal and varicocele was investigated in table II. A significant positive relationship was observed between sperm concentration (r = 0.673, p < 0.001), motility percentage (r = 0.492, p < 0.001), and sperm morphology (r = 0.742, p < 0.001) with ATP. Also, a significant negative relationship between the percentage of DNA fragmentation and ATP (r = -0.466, p < 0.001) was observed.

**Figure 3:**
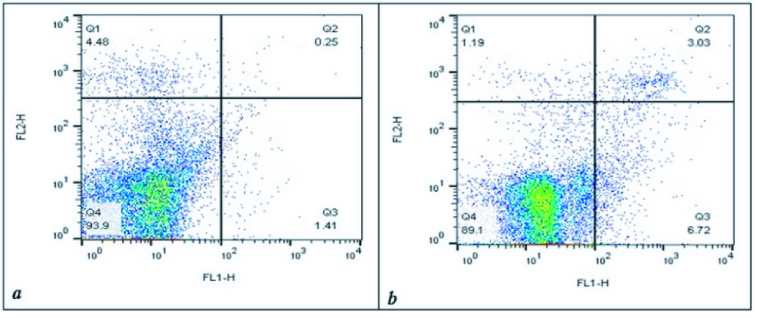
Apoptosis expression levels between (a) normal and (b) varicocele group. The lower right quadrants (Q3) represented the percentage of early apoptotic sperms and the upper right quadrants (Q2) represented the percentage of late apoptotic sperms. The overall sum of apoptotic sperm was Q2+Q3 = 5.7 ± 4.5 in the normal group and Q2+Q3 = 8.1 ± 6.97 in the varicocele group.

Apoptosis assay in human sperm between the 2 groups was performed by Annexin V-FITC. The PS in apoptotic sperm transfers to the outside of the membrane and then the percentage of the apoptotic sperm can be estimated by PS-Annexin V conjugation complex detection. The result of one sample in each group was demonstrated in figure 3. No significant difference was observed during apoptosis between varicocele and the normal group (p < 0.001), table I. The relationship between sperm parameters and the percentage of apoptosis in normal and varicocele was investigated in table II. There was a significant negative relationship between sperm concentration (r = -0.334, p < 0.001), motility percentage (r = -0.334, p < 0.001), and normal sperm morphology (r = 0.328 p < 0.001) with apoptosis, but in the varicocele group, no correlation was observed between apoptosis and DNA fragmentation (r = 0.029, p = 0.77).

**Table 1:** Comparison of the conventional semen parameters analyses, sperm DNA fragmentation, MMP, ATP, and apoptosis between 2 groups


**Parameters**	**Control (n = 50)**	**Varicocele (n = 50)**	**P-value**
**Concentration (million/m)**	53.9 ± 17.5	22.5 ± 10.8	< 0.001
**Motility (%)**	40.6 ± 8.03	27 ± 8.5	< 0.001
**Morphology (%)**	3.7 ± 0.94	1.2 ± 0.58	< 0.001
**DNA fragmentation index (%)**	18.9 ± 4.4	26.2 ± 5.1	< 0.001
**Mitochondrial membrane potential (%)**	82.8 ± 15.1	38.2 ± 10.4	< 0.001
**Adenosine triphosphate**	1.3 ± 0.20	0.98 ± 0.08	< 0.001
**Apoptosis (%)**	5.7 ± 4.5	8.1 ± 6.97	0.055
Data were presented as Mean ± SD. Mann-Whitney U test, MMP: Mitochondrial membrane potential, ATP: Adenosine triphosphate			

**Table 2:** Spearman's correlation coefficient between DFI, MMP, ATP and apoptosis with semen parameters


**Variables**		**Parameters**	**P-value**
**DFI**			
	Correlation coefficient	-0.50 ${}^{**}$
	**Concentration**	Sig. (2-tailed)	< 0.001
		Correlation coefficient	-0.37 ${}^{**}$
	**Motility**	Sig. (2-tailed)	< 0.001
		Correlation coefficient	-0.61 ${}^{**}$
	**Morphology**	Sig. (2-tailed)	< 0.001
		Correlation coefficient	0.80 ${}^{**}$
	**Concentration**	Sig. (2-tailed)	< 0.001
		Correlation coefficient	0.67 ${}^{**}$
	**Motility**	Sig. (2-tailed)	< 0.001
		Correlation coefficient	0.71 ${}^{**}$
	**Morphology**	Sig. (2-tailed)	< 0.001
		Correlation coefficient	-0.41 ${}^{**}$
	**DFI**	Sig. (2-tailed)	< 0.001
**ATP**			
	Correlation coefficient	0.67 ${}^{**}$
	**Concentration**	Sig. (2-tailed)	< 0.001
		Correlation coefficient	0.49 ${}^{**}$
	**Motility**	Sig. (2-tailed)	< 0.001
		Correlation coefficient	0.74 ${}^{**}$
	**Morphology**	Sig. (2-tailed)	< 0.001
		Correlation coefficient	-0.46 ${}^{**}$
	**DFI**	Sig. (2-tailed)	< 0.001
**Apoptosis**			
	Correlation coefficient	-0.33 ${}^{**}$
	**Concentration**	Sig. (2-tailed)	< 0.001
		Correlation coefficient	-0.33 ${}^{**}$
	**Motility**	Sig. (2-tailed)	< 0.001
		Correlation coefficient	-0.32 ${}^{**}$
	**Morphology**	Sig. (2-tailed)	< 0.001
		Correlation coefficient	0.02
	**DFI**	Sig. (2-tailed)	0.77
Data were presented as Spearman correlation. Rho: Rank correlation coefficient, **Correlation is significant at the 0.01 level (2-tailed). DFI: DNA fragmentation index, MMP: Mitochondrial membrane potential, ATP: Adenosine triphosphate			

## 4. Discussion

The pathogenesis of varicocele is not clearly defined, but the proposed mechanisms by which male fertility is impaired by varicocele are mainly attributed to DNA damage, apoptosis, and OS. Other factors such as hypogonadism in adolescence (i.e., lowering in follicle-stimulating hormone), androgen binding protein, transferrin, and inhibin), reflux of toxic metabolites (e.g., cadmium accumulation), scrotal hyperthermia, and hypoxia all approve this theory that high testicular temperature, will destroy the spermatogenesis in cellular and molecular level and impair the testis and Leydig cell function (15). The association between morphological and functional alteration of testis and varicocele was first considered by Johnsen and Agger, who demonstrated the improvement of testis biopsy results before and after varicocele operation (i.e., from the appearance of seminiferous tubules after surgery and increase of the germ cells concentration) can improve the testicular function (16).

The growth in our knowledge of the cellular and molecular aspects of varicocele pathogenesis has provided a new therapeutic area for physicians to treat varicocele. Involvement of testicular heat stress, involvement in potential hypoxia, and inflammation subsequently result in a high level of OS as a main pathogenetic mechanism in infertile men with varicocele that decreases their semen quality and fertility (6).

Increase in free radicals due to overproduction of ROS in combination with lowering of antioxidant level results in OS shock in turn. This condition facilitates nuclear DNA damage, mitochondrial DNA gene alterations, and many other probability pathological events that are triggered by poor sperm quality. There is an association between sperm chromatin damage, sperm DNA fragmentation, and, OS with semen parameters quality/spermatogenesis. Taken together, all of the mentioned processes may result in high levels of sperm mitochondrial inactivity, sperm apoptosis and sperm DFI.

Our purpose in the present study was to illuminate that MMP and ATP contents assessments are essential tests for evaluating sperm mitochondrial function. They significantly correlate with DFI and apoptosis and also with semen parameters.

In this study, sperm parameters such as sperm concentration, motility, and sperm morphology were reduced in varicocele compared to normal subjects. This is consistent with the results of Mongioì and co-workers that represent the negative impact of varicocele on the quality of sperm parameters before the repairing operation (17). Also, the present study showed that DNA fragmentation increased in the sperms of the varicocele group compared to the control group. This is consistent with the results of Dieamant et al., which reported that varicocele increases the level of sperm DNA fragmentation (18).

Observation of significantly higher levels of ROS usually is associated with DNA damage, apoptosis, and OS. On the other hand, increasing free radicals due to overproduction of ROS in combination with lowering of antioxidant level results in switching on of the OS circuit in turn. This condition facilitates nuclear DNA damage, mitochondrial DNA gene alterations, and many other probability pathological events that are triggered by poor sperm quality. There is an association between sperm chromatin damage, sperm DNA fragmentation, and, os with semen parameters quality/spermatogenesis. It was shown that varicoceles impair the semen parameters through all of the mentioned mechanisms.

Sperm motility is achieved through 2 main metabolic pathways in the form of ATP production, glycolysis in the cytoplasm and OXPHOS in mitochondria. Both metabolic pathways are active for energy production and motility in sperm. The production of OXPHOS-derived ATP appears to be more important for sperm function than sperm motility. Electrons from pyruvate catalysis and β-fatty acid oxidation metabolism in the TCA cycle are gradually transferred to O_2_ via electron transport chain complexes, generating potential in the healthy mitochondrial membrane by producing a proton (H+) gradient, which is determined by MMP assay.

Mitochondria are responsible for providing energy for the movement of the sperm flagella, capacitation, zona penetration, and sperm functionality. Therefore, they play a very important role in fertilization. Also, there is evidence of a strong association between mitochondrial function and semen parameter quality consistent with our results. Mitochondrial dysfunction may lead to electron leakage, increased ROS, OS, and decreased MMP, which in turn can lead to decreased ATP production (19). Therefore, we proposed the MMP evaluation as an important indicator of sperm mitochondrial function. We have proven a strong correlation between MMP and ATP contents with sperm quality and DFI, which indicates sperm fertilizing potential (20).

The present study shows that the varicocele has a negative effect on sperm concentration, motility, morphology, sperm DFI, MMP level, and ATP. However, no significant apoptosis was observed in the sperm of varicocele individuals compared with normal. Data analysis showed that MMP and ATP assays are 2 important tests as a biomarker to evaluate mitochondrial function. It can be used for diagnosis and treatment of infertile men indicating that healthy sperm can be implemented in ART methods as a sperm functionality test.

The absence of a significant correlation between apoptosis and varicocele in this study may be due to the small sampling. However, observing a significant relationship between varicocele and reducing the quality of sperm parameters and DFI on one side, simultaneously with observing a significant correlation with the MMP and ATP contents indicates a serious consequence on sperm mitochondrial function, which may be reflected in sperm function and fertility. Therefore, it is recommended to pay special attention to the assessment of sperm mitochondrial function, including MMP, and ATP content assay as a sperm functional test.

## 5. Conclusion

The present study shows that the varicocele has a negative effect on sperm concentration, motility, morphology, sperm DFI, MMP level, and ATP. But there was no significant apoptosis in the sperm of varicocele individuals compared with normal. Data analysis showed that MMP and ATP assays are 2 important tests to evaluate mitochondrial function. It can be used as a diagnosis and treatment of infertile men indicating that sperm healthy can be implemented as a sperm functionality test in ART methods.

##  Conflicts of Interest

There is no conflict of interest in this study.
